# Neuro-Immune Regulation in Inflammation and Airway Remodeling of Allergic Asthma

**DOI:** 10.3389/fimmu.2022.894047

**Published:** 2022-06-16

**Authors:** Ning Zhang, Jing Xu, Congshan Jiang, Shemin Lu

**Affiliations:** ^1^ National Joint Engineering Research Center of Biodiagnostics and Biotherapy, Second Affiliated Hospital, Xi’an Jiaotong University, Xi’an, China; ^2^ Key Laboratory of Environment and Genes Related to Diseases, Xi’an Jiaotong University, Ministry of Education, Xi’an, China; ^3^ Institute of Molecular and Translational Medicine (IMTM), Xi’an Jiaotong University Health Science Center, Xi’an, China; ^4^ Department of Biochemistry and Molecular Biology, School of Basic Medical Sciences, Xi’an Jiaotong University Health Science Center, Xi’an, China; ^5^ National Regional Children’s Medical Center (Northwest), Key Laboratory of Precision Medicine to Pediatric Diseases of Shaanxi Province, Xi’an Key Laboratory of Children’s Health and Diseases, Shaanxi Institute for Pediatric Diseases, Xi’an Children’s Hospital, Affiliated Children’s Hospital of Xi’an Jiaotong University, Xi’an, China

**Keywords:** neuro-immune regulation, inflammation, airway remodeling, asthma, pulmonary neuroendocrine cells

## Abstract

Allergic asthma is a common chronic inflammation of the airways and causes airway remodeling eventually. For a long time, investigators have been focusing on the immunological mechanism of asthma. However, in recent years, the role of neuro-regulation in the occurrence of asthma has gradually attracted investigators’ attention. In this review, we firstly describe neuro-immune regulation in inflammation of allergic asthma from two aspects: innate immunity and adaptive immunity. Secondly, we introduce neuro-immune regulation in airway remodeling of asthma. Finally, we prospect the role of pulmonary neuroendocrine cells in the development of asthma. In general, the amount of researches is limited. Further researches on the neural regulation during the occurrence of asthma will help us clarify the mechanism of asthma more comprehensively and find more effective ways to prevent and control asthma.

## Introduction

Allergic asthma is a chronic inflammation of the airways caused by repeated exposure to allergens such as dust, mites and pollens (1). It is a disease with high heterogeneity (2). The hallmark of asthma is airway hyperresponsiveness (AHR) with symptoms of cough, shortness of breath, wheeze and chest tightness. Asthma is a serious global problem with the prevalence rate of 5.8% in children under 18 and 8.4% in adults, and fatality rate of 1.3/100,000 (https://www.cdc.gov/nchs/fastats/asthma.htm [Accessed March 8, 2022]). Asthma can be divided into Type 2-High and Type 2-Low subtypes according to whether T helper 2 (Th2) cells are dominant (3). Type 2-High asthma, also known as allergic asthma, is characterized by large numbers of Th2 cells and group 2 innate lymphoid cells (ILC2s). By secreting type 2 cytokines, these two types of cells can initiate a series of signaling cascades (4–6). It starts with the trigger of allergen which is captured by antigen-presenting cells such as dendritic cells. Then Th2 cells in lymph nodes are induced and activated (7, 8) with an antigen specific manner. In addition, injured epithelial cells release cytokines such as IL-25, IL-33, and thymus stromal lymphopoietin (TSLP), which can directly recruit and activate Th2 cells and ILC2s in an antigen-nonspecific manner (9–11). These two types of cells produce a large number of type 2 cytokines and activate effector cells such as B cells, basophils and eosinophils, thus initiate the pulmonary inflammatory response (12, 13), causing pathological changes such as AHR, excess mucus secretion, and airway remodeling (14).

Although immune response plays a vital role in the occurrence and development of allergic asthma, neuro regulation, especially neuro-immune regulation in asthma has drawn more and more attention in recent years. Just as skin and intestine, the lung is an important organ that connects the body with the environment. Airway epithelial cells are exposed to all kinds of external irritants from air sources during breath. The lung is the first organ to sense and recognize dangers by alarming the body immediately and responding to these foreign invaders. During this process, nervous and immune system cooperate closely. They share many similarities, such as the same universal distribution almost all over the body, common transmitting mediators such as neurotransmitters and cytokines. In addition to exercising their basic innate defense function, the innate nervous/immune system of the body can also continuously promote adaptive regulation with the changes of external environment (15). Nervous and immune system interact with each other. Sensory nerve fibers express cytokine receptors, which can sense cytokines and send messages to the brain *via* the autonomic nervous system (15–17). Neurotransmitters can also regulate immune responses through neural receptors on the surface of immune cells (18, 19). This review focuses on neuro-immune regulation in inflammation and airway remodeling of allergic asthma.

## Neuro-Immune Regulation in Inflammation of Allergic Asthma

The lung is a highly innerved organ. Nerves in the lung can be divided into sensory or afferent nervous system and motor or efferent nervous system according to the signal direction travelling within the nerve (20, 21). Sensory nerves from the airways relay stretch stimuli, mechanoreceptors, and chemical stimuli, chemoreceptors, along afferent sensory fibers or vagus nerves to the central nervous system (20). Sympathetic, parasympathetic, non-adrenergic and non-cholinergic parasympathetic nerves constitute the motor pathways of the lung. The parasympathetic nerve releases acetylcholine (ACh) and activates muscarinic M3 receptors on airway smooth muscle cells (ASMs), resulting in the effects of bronchial contraction (22, 23). The pulmonary sympathetic nerve innervates the blood vessels and submucosal glands of the bronchus and antagonizes parasympathetic action by noradrenaline (24). Non-adrenergic non-cholinergic parasympathetic nerves may mediate bronchiectasis through vasoactive intestinal peptides and nitric oxide (25).

### Neuro-Immune Regulation in Innate Immune Cells

**Figure 1:**
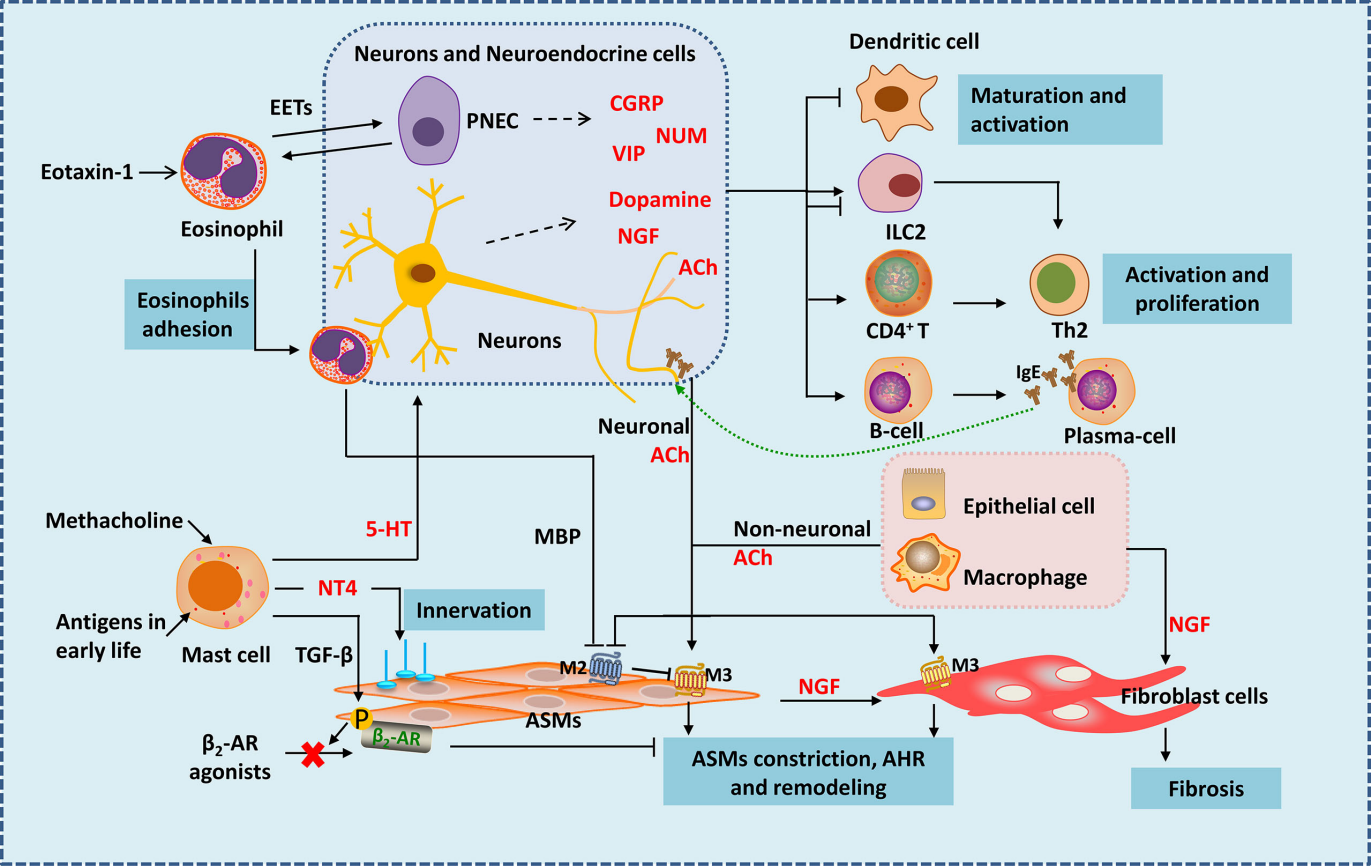
Neuro-immune interactions in inflammation and airway remodeling of allergic asthma. Eosinophils migrate to airway during inflammation *via* eotaxin-1. They release EETs surrounding and activating PNECs. Under the action of VLA-4 and CD11b, eosinophils adhere to VCAM-1 and ICAM-1 on parasympathetic fibers. Eosinophils are then activated and release MBP, which is an antagonist of M2 muscarinic receptor, thus enhancing parasympathetic mediated bronchoconstriction. Besides, eosinophils increase airway innervation. Under the stimulation of methacholine, mast cells release 5-HT, interacting with 5-HT_2_ receptors in parasympathetic nerves, thus release ACh (neuronal ACh), enhancing bronchoconstriction. Mast cells also produce NT4 following allergen exposure during early-life, increasing ASMs innervation through NT4/TrkB signaling, causing long-term airway dysfunction. Mast cells also produce TGF-β to induce β2-AR phosphorylation in ASMs, thereby causing β2-AR agonists resistance. Neuropeptides generate form neurons and neuroendocrine cells such as NMU and VIP activate ILC2s. CGRP generated mainly from PNECs inhibits maturation of DCs, it may have bidirectional effects on ILC2s. Besides neuronal ACh, non-neuronal ACh released from epithelia cells and macrophages mainly acted in small airways. ACh activate muscarinic 3 acetylcholine receptor on ASMs and fibroblast cells, causing airway contraction and airway remodeling. NGF released from neurons, epithelial cells, ASMs and other immune cells acts on fibroblasts leading to fibrosis. NGF can also activate Th2 cells and promote the differentiation of B cells into plasma cells. EETS, Eosinophil extracellular traps; PNEC, pulmonary neuroendocrine cell; MBP, major basic protein; 5-HT, 5-hydroxytryptamine; ACh, acetylcholine; NT4, neurotrophin 4; ASMs, airway smooth muscle cells; NGF, nerve growth factor.

Like a guard, dendritic cells (DCs) are the first to sound the alarm when allergens invade. Allergen capture by antigen-presenting cells such as DC cells is the beginning of allergic asthma (7, 8). Nervous system regulates DCs by neuropeptides. Calcitonin gene-related peptide (CGRP) is a neuropeptide secreted by pulmonary neuroendocrine cells (PNECs) mainly in lung. It inhibits maturation of DCs by CGRP receptors expressed in DCs themselves, reducing the activation and proliferation of antigen-specific T cells (**Figure 1**). In mouse model of ovalbumin (OVA) induced asthma, CGRP-pretreated DCs alleviate inflammation of allergic asthma with reduced eosinophil numbers in bronchoalveolar lavage fluid (26). Interestingly, the nerve cells can also be a whistleblower to allergens thus giving instructions to DCs. A study using skin allergen exposure model found that allergens can directly activate transient receptor potential vanilloid 1 positive sensory neurons in skin, causing itch and pain behaviors. Activated neurons release neuropeptides such as substance P, which can induce the migration of CD301b^+^ DCs into the draining lymph nodes, where the differentiation of Th2 cells is initiated (27). Whether the nervous system is also the first responder of allergens during asthma, thereby triggering the accumulation of dendritic cells and subsequent immune responses needs further exploration. If so, inhibition of sensitization by the allergen to sensory nerve fibers could potentially reduce the activation of pulmonary inflammation during asthma. Besides, antigens can also lead to the variation of innervation, regulating inflammatory infiltration in tissues in an age-related manner. In neonatal mice, exposure to antigens could elevate neurotrophin 4 (NT4) levels of ASMs thus increase ASMs innervation through NT4/TrkB signaling. As a result, persistent AHR appears in adulthood (28) (**Figure 1**). Therefore, in addition to the familiar antigen-presentation process, the interaction between the nervous system and antigens might be more important during the initial phase of inflammation.

In asthma, mast cells accumulate within or close to epithelium and smooth muscle under the stimulation of allergen (29). Mast cells could mediate acute inflammatory response of asthma by secreting a large number of pro-inflammatory and pro-airway constrictor mediators (29–32). Mast cell is now recognized to be involved in nerve regulation during the development of asthma—with muscarinic M3 receptors on its surface (33). Bronchial stimulation test by methacholine provocations is a classic method used for clinical diagnosis of asthma. Mast cells may play the key role in this response. Under the stimulation of methacholine, mast cells release 5-hydroxytryptamine (5-HT), interact with 5-HT2 receptors in parasympathetic nerves, thus release ACh (**Figure 1**). ACh, an endogenous neurotransmitter, enhances bronchoconstriction and AHR in house dust mite induced experimental asthma (33). Therefore, mast cells and parasympathetic neurons cooperate to form a vicious circle that amplifies the bronchial constriction of choline. Asthma gets worse as the result. Why are mast cells involved in parasympathetic regulation of ASMs? Why the parasympathetic nerve manipulates mast cells to enhance its control on ASMs? Is it the result of the body’s rapid response to foreign substances? Studies showed that the interference of mast cells in ASMs occur with the development of ASMs. Following allergen exposure during early-life, mast cells in lung can produce amount of NT4, which is essential for the innervation of ASMs during development (34), causing long-term airway dysfunction. Mast cells can also produce transforming growth factor β (TGF-β) to induce β2-adrenergic receptor (β_2_-AR) phosphorylation in ASMs, thereby reducing the airway dilation effect of β_2_-AR agonists (35) (**Figure 1**). Therefore, during the occurrence of allergic asthma, mast cells are activated by immunity and affect ASMs under nerve regulation, resulting in persistent airway contraction and AHR. Obviously, mast cells are closely related to the nervous system. Mast cells seem to control the distribution and function of nerves. Besides inflammatory factors, are mast cells also regulated by neurotransmitters? After all, signals travel much faster in nerves. More research is expected.

Eosinophil is accepted as an important kind of effector cells in the development of allergic asthma. Extensive eosinophil infiltration around the airway is considered a hallmark of allergic asthma (36–38). However, growing evidence suggests that eosinophils are inextricably linked to the nervous system during the development of asthma. Tropomyosin receptor kinase A is a high affinity receptor for nerve growth factor (NGF). In a tropomyosin receptor kinase A-knock-in mouse model, eosinophils are found to migrate to airway during inflammation *via* eotaxin-1 (39). Moreover, eosinophils are closely related to nerve fibers in asthma (40, 41). Under the action of VLA-4 and CD11b, eosinophils adhere to VCAM-1 and ICAM-1 on parasympathetic fibers (41) (**Figure 1**). It was found that in guinea pigs (42) and monkeys (43) during allergy, eosinophil binding to VCAM-1 or ICAM-1 is important in airway hyperreactivity. Eosinophils are then activated and release major basic protein (MBP), which is an antagonist of M2 muscarinic receptor in human, thus enhancing parasympathetic mediated bronchoconstriction (40) (**Figure 1**). Besides, airway and peripheral blood eosinophils are associated with increased airway innervation in asthma patients. Eosinophils increase airway sensory innervation in mice and humans, which is remarkable in moderate persistent asthma, thus leading to AHR (44). Strikingly, a novel study suggests that eosinophils play a much more interesting role in neuro-immune regulation than we previously have known. Eosinophil extracellular traps (EETs) are web-like DNA traps generated from eosinophils (**Figure 1**). In asthma tissues from human and mice, PNECs are surrounded by EETs and activated *via* the CCDC25–ILK–PKCα–CRTC1 pathway (45). It appears that PNECs are one type of the target cells of eosinophils, but previous studies have shown that eosinophils are influenced by cells such as PNECs (46). Such interactive network regulation is not unusual for sophisticated organisms like mammals, and suggests that we need to take a dialectical view of disease.

ILC2s are important players in both neural and immune regulation. They play a vital role in asthma by releasing type 2 cytokines. Besides respond to alarmins IL-25, IL-33 and TSLP which are released by epithelium, ILC2s could also be regulated by other stimuli. ILC2s are bidirectional regulated by the nervous system (**Figure 1**). *In vitro*, *N*mur1 is highly expressed in ILC2s purified from mice, and neuropeptides neuromedin U (NMU) activate ILC2s by interacting with NMUR (18, 47, 48). In house dust induced airway inflammation model, NUM amplifies allergic inflammation with the combination of IL-25 (18). Vasoactive intestinal peptide (VIP) is another neuropeptide that belongs to non-adrenergic, non-cholinergic (NANC) system. VIP is one of the most potent endogenous bronchodilators and is proposed to have anti-inflammatory effects (49). However, recent study find it expressed at high levels in nodose ganglion neurons in the lung and has a positive stimulating effect on ILC2. Under the stimulation of IL-5 and OVA, the generation of VIP increase in nodose ganglion neurons both *in vitro* and *in vivo*. VIP may promote ILC2s activation partly by VIP-VPAC2 axis thus amplify the airway inflammatory (50). ILC2s are found resided in proximity to PNECs in naïve mice, and when cultured with CGRP and IL-33, ILC2s are activated with increased IL-5 production (46). However, another study analyzed single-cell RNA-seq atlas of lung ILCs and found that ILC2s express both CGRP and its encoding genes, and endogenous CGRP derived from neurons and neuroendocrine cells could negatively regulate IL-33 or IL-25 driven pulmonary ILC2s response, inhibit the production of ILC2-derived type 2 cytokines, thus reduce tissue damage under certain conditions (19). In a study of Nippostrongylus brasiliensis infection mouse model also showed that a subset of ILC2s in lung express CGRP receptor components, and CGRP limits ILC2s response and worm clearance (51). This suggests the complexity of CGRP function and ILC2s microenvironment in different disease models. Many neuro-derived factors can inhibit type 2 immune responses mediated by ILC2s. β_2_-AR is an important member of the adrenergic nervous system. β_2_-AR gene is detected in murine and human ILC2s. In *N. brasiliensis* and *Alternaria alternata* induced murine lung inflammation models, ILC-deficient mice exhibit increased levels of ILC2s in the lung after infection, which could be inhibited by β_2_-AR agonist treatment (52). β_2_-AR agonist is an important class of medicine in the treatment of asthma. It can reduce bronchial constriction by targeting β_2_-AR on ASMs. Whether β_2_-AR signaling also alleviate asthma symptoms by dampening ILC2 responses needs further study. α7-nicotinic acetylcholine receptor (α7nAChR) is thought to have anti-inflammatory effect in inflammatory diseases (53). α7nAChR is found expressed on murine ILC2s and α7nAChR agonist inhibited ILC2s function with decreased IL-5 and IL-13 production *in vitro*. By giving intranasal recombinant mouse IL-33 to Rag2-deficient mice (devoid of T and B cells), ILC2s are induced and a7nAChR agonist treatment could ameliorate ILC2s-mediated AHR by decreasing the expression of GATA-3, a key transcription factor of ILC2s (54). In conclusion, ILC2s express a variety of neuropeptide receptors. As the innate immune cells in the lung, ILC2s become the pivot of lung neuro-immune regulation.

We know that there are other types of innate immune cells, like macrophages, basophils and neutrophils. A special group of macrophages have been found near the large bronchi and airway nerves, named nerve-and airway-associated macrophages (55). This kind of macrophages can activate neurons to produce colony stimulating factor 1 (CSF1) by producing bone morphogenic protein 2, and CSF1 is the necessary signal to maintain its survival. So, nerve fibers provide nutrients for the survival of macrophages. But researches on their relationship with nervous system is still insufficient.

### Neuro-Immune Regulation in Adaptive Immunocytes

As we mentioned above, large numbers of Th2 cells is a hallmark of Type 2-high asthma. Th2 cells, induced and activated in lymph nodes by DCs in an antigen specific manner, produce large amounts of type 2 cytokines thus activate further immune cascades. Stimulated by Th2 cells, B cells mature into plasma cells and secrete IgE in response to cytokines IL-4 and IL-13 (56). IgE interacts with cells that have receptors FcϵRI and CD23, such as mast cells, basophils, DCs and ASMs, causing acute allergic response and AHR (3, 57).

Differentiation of T cells into Th2 cells is a landmark in the development of allergic asthma, and the subsequent production of large amounts of type 2 cytokines cause persistent inflammatory changes. These are also important molecular indicators for determining asthma severity and evaluating the effectiveness of interventions in various studies. T cells are certainly an important battleground for the nervous system to participate in immune regulation. As a transient receptor potential ankyrin channel, transient receptor potential ankyrin 1 (TRPA1) is widely expressed in sensory neurons. It can be triggered by wide variety of stimuli and release amounts of inflammatory neuropeptides and neurotransmitters, facilitating the communication between the nervous system and the immune system (58). Furthermore, adaptive immune cells may directly participate in the battlefield of neuroimmune response through TRPA1. The expression of TRPA1 is increased in lung tissues and CD4^+^ T cells in an OVA-induced mouse model of asthma, accompanied with the development and exacerbation of asthma (59). It provides a complement to non-neural sources of TRPA1. As we have discussed before, the neurotransmitter VIP regulates the immune system by acting on its receptor VPAC2. With the help of VPAC2 expressed on T cells, VIP promotes the differentiation of CD4^+^ T cells into Th2 cells and increase the production of IL-5 and IL-13 correspondingly (50). NGF has been suggested to play an important role in neuro-immune regulation in airway inflammation by overwhelming studies (60–62). The aggravating role of NGF in lung inflammation can be explained partly by regulating T and B cells. NGF exacerbates inflammation and airway remodeling by enhancing Th2 in OVA sensitized asthma in rat model (63). Suppression of NGF could reduce Th2 immune response, decrease inflammatory infiltration and airway response in murine asthma model (64). The above effects in lung inflammation resulting from inhibition of NGF is also observed in mice infected with respiratory syncytial virus (RSV) (65). And RSV infection in infants is considered a risk factor for the development of asthma (66, 67). It seems that NGF is a broad and powerful molecule involved in neuroimmune inflammation. It is worth noting that the regulation of adaptive immunity by the nervous system may partly explain the higher incidence of allergic asthma in children. It is found that during postnatal development in mice and humans, sympathetic nerves in the lung in mice and humans undergo a transition from dopaminergic type to adrenergic type. In this process, dopamine is bound to a specific dopamine receptor, DRD4, then IL-2-STAT5 signaling is upregulated and histone trimethylation is induced at Th2 gene loci, as a result, differentiation of CD4^+^ T cell to Th2 cell is promoted (68). Thus, the dopamine-DRD4 pathway augments Th2 inflammation in the lung of young mice in allergen exposure models, which is not so evident in the lungs of adult mice dominated by the adrenergic type.

NGF can also affect the function of plasma cells in the development of asthma. In a mouse model of OVA induced allergic asthma, plasma cells from airways and spleen express different patterns of neurotrophins receptors, and TrkA is only expressed on pulmonary plasma cells. *In vitro*, NGF promotes survival of pulmonary plasma cells by increasing transcription factors in plasma cells (X-box binding protein 1 and NF-κB subunit RelA) responsible for production of immunoglobulins. Consistently, anti-NGF treatment reduce the number of pulmonary plasma cells and serum IgE (69). B cell activation and subsequent production of large amounts of antigen-specific IgE are known to be characteristic of allergic asthma. However, studies on neural regulation on B cells or plasma cells in asthma are handful and years in advance. Interestingly and excitingly, FcϵR1, a high-affinity IgE receptor, is found expressed on vagal nociceptor neurons in lung in an OVA sensitized mouse model. It means that this kind of neurons can sense invading allergens directly, inducing allergic inflammation (70). This finding is an important addition to the understanding of neuro-immune regulation in the development of asthma, and more research is expected.

## Neuro-Immune Regulation in Airway Remodeling of Allergic Asthma

Airway remodeling, the ultimate pathological change in the development of many pulmonary diseases, is one of the most characteristic pathological features of persistent asthma and main reason for hospital care (3, 71). ASMs and fibroblasts play important roles in airway remodeling, and they are closely related to the nervous system.

ASMs express receptors for IgE and respond to IgE directly, causing airway obstruction in severe asthma (3, 72). ASMs is the main kind of effector cell of AHR and airway remodeling, especially in small airways (73). ASMs becomes innervated by parasympathetic fibers since embryogenesis, and its development partly rely on ACh secreted from parasympathetic nerves (74). In asthma, the airway tone is increased mainly because of ASMs contraction, induced by ACh (**Figure 1**). ACh is derived from two ways——neuronal (the classic) and non-neuronal way. The former is released from nerves like vagus and parasympathetic nerves, the latter comes from epithelial cells and macrophages, which is the main source in small airways (75). ACh activated muscarinic 3 acetylcholine receptor on ASMs and fibroblast cells, causing airway contraction and airway remodeling (75, 76). It is unclear whether the ACh from these two sources is different. Besides, just as we discussed, eosinophils increase parasympathetic ACh release by inhibiting M2 receptor function. An increase in airway eosinophils specifically enhances bronchial constriction in mice, independent of changes in muscarinic M3 receptors or 5-HT receptors in ASMs (77). Apparently, the body considers neuromodulation to be an efficient and economical way. Even without the involvement of nerve fibers, various cells of the body communicate with each other through neurotransmitters. During evolution for over tens of millions of years, human beings and other mammals have developed such elaborate structures. Making full use of the intrinsic transmitters and receptors not only enables us to respond quickly to the environment, but also is more cost-effective——far more efficient and economic than evolving one or more extra regulatory systems.

Excessive collagen deposition and subepithelial fibrosis is another striking feature of airway remodeling (78–81). Almost all these mechanisms are associated with the activation of fibroblasts and its trans-differentiation into myofibroblasts (82, 83). TGF-β1 is identified as one of the important factors in this process (84). However, more and more researches reveal that neural regulation also plays a vital role (**Figure 1**). Previous studies have shown that NGF was highly increased during allergic asthma, especially in chronic phase (85). And NGF induced the production of type III collagen in fibroblasts by activating p38 MAPK in a TGF-β1 independent way, thus promoting fibrosis (85). Interestingly, activated airway epithelium is a major source of NGF in allergic airway inflammation (86). And NGF can also be produced by ASMs and infiltrated immune cells including mast cells, T cells, eosinophils (87–90). It seems that when a man is going down-hill, everyone will give him a push.

Besides, macrophage may promote the development of pulmonary fibrosis (91) by affecting nerves in the lung. In a bleomycin inhaled mouse model, macrophage-derived neuronal guidance proteins such as netrin-1 is involved in pulmonary adrenergic nerve remodeling and promotes pulmonary fibrosis (92).

## Regulation of PNECs in Allergic Asthma

PNECs are a rare and evolutionarily conserved type of lung epithelial cells, accounting for about 0.5% of airway epithelial cells (93), 0.01% of total lung cells (94). They were firstly described in 1954 (95). They scatter among lung epithelial cells, or exist in clusters, forming neuroepithelium bodies (NEBs) consisting of 5-20 PNEC cells (96–98). PNECs are located along the airway epithelium in the trachea and lung in humans and rodents, while NEBs are mainly distributed at the airway branch points (96), where inhalation particles gathered (99). PNECs originate from the endoderm or basal cells (100–102). PNECs are the first to differentiate in the lung (96). The differentiated PNECs gradually move towards the bronchial bifurcation to form NEBs. Nerve fibers then enter the NEBs to innervate them, and innate lung immune cells such as ILC2 settle around the NEBs (96). Although the population of PNECs is very limited, their function can be significant. PNECs have both neural and endocrine properties. They are the only innervated cells in the lung epithelium and the cytoplasm of PNECs is rich in core vesicles containing many kinds of substances like amines, amine metabolizing enzymes, purines, neuroendocrine markers, functional proteins and so on (103). *In vitro*, PNECs release their vesicle contents under the stimulation of oxygen, mechanical stretching and chemical spines, thereby inducing corresponding pathophysiological changes (104). Innervation of PNECs appear in rabbit embryos on the 16th day (105). The specific form of PNECs and nerve interaction varies in different species (96), which is still not well understood. Present study shows that the nerve innervating PNECs may be sensory nerve, and its cell body is located in the vagal ganglion or dorsal root ganglia (106). PNECs are considered as the most important “sense” cells in the airways. They can sense oxygen changes in the airways (107). The number of PNECs increases in hypoxic environment, and plays a protective role in the surrounding airway epithelial cells (100). Some members of the olfactory receptor family are found expressed in human PNECs, thus PNECs can sense a variety of chemical stimuli (108). Besides, they can also sense mechanical stimuli (109, 110). When the airway epithelium is damaged, PNECs act as progenitor cells to repair the damaged epithelium (97, 111, 112). PNECs are not the key cells to maintain lung development (46), but are necessary in many lung diseases including asthma (46, 113–115).

**Figure 2:**
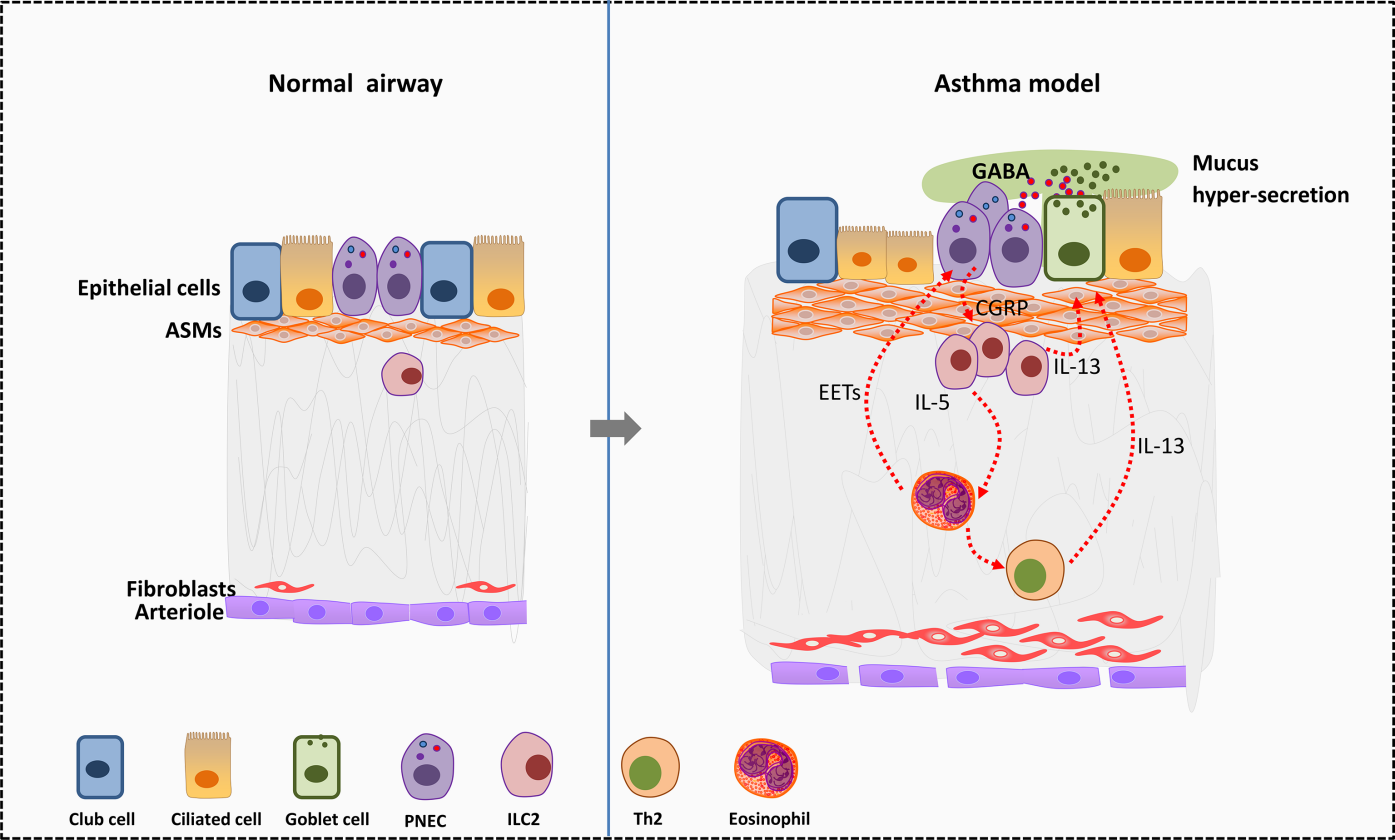
Effects of PNEC cells on surrounding cells in allergic asthma. In murine asthma model, PNECs secret CGRP to active ILC2s and further promote the differentiation of Th2 cells. Type 2 cytokines such as IL-5 and IL-13 secreted by ILC2s act on eosinophils and goblet cells. Eosinophils in turn activate PNECs by releasing EETs. Besides, PNECs secret GABA to promote transformation of club cells near PNECs into goblet cells. GABA leads to excess mucus secretion in airway goblet cells by acting on GABA type α and GABA type β receptors, thus worse symptoms in asthma. EETS, Eosinophil extracellular traps; PNECs, pulmonary neuroendocrine cells; GABA, gamma-aminobutyric acid; ASMs, airway smooth muscle cells.

PNECs may serve as the center of neuro-immune regulation in the asthmatic lung (116). PNECs activate ILC2s by secreting neuropeptides thus amplify the allergen stimulation of immune cells in asthma (46). PNECs are the main source of gamma-aminobutyric acid (GABA) in the lung. GABA secreted by PNECs can promote the transformation of club cells around PNECs into goblet cells. In addition, GABA leads to excess mucus secretion in airway goblet cells by acting on GABA type α and GABA type β receptors (117) and worse symptoms in asthma. Adjacent to ILC2s, PNECs can directly stimulate ILC2s to produce IL5, IL-13 and other cytokines by secreting CGRP, and then trigger Th2 response (46) (**Figure 2**).

## Conclusion

The occurrence of asthma involves multi-factors such as heredity, environment and so on. Neuro-immune regulation is ubiquitous in the development of asthma and airway remodeling. The link between the nervous system and the immune system is intricate and interlocking. This review provides a new view for the study of the pathogenesis of allergic asthma and the search for effective treatment from the perspective of nerve regulation on immunity. The nervous system is closely related to the various cells of the immune system, but it is not clear whether neuro-immune regulation is the cause or the result for the development of asthma. With the discovery of PNECs, the function of neuro-regulation in the physiological and pathological aspects in asthma has been paid more attention gradually. However, related investigations are still very handful. There are still many questions to be answered. For example, most of the signaling pathways and key molecules that mediate neuro-immune regulation are not yet clear, and the function of some cells and molecules is controversial.

With the novel perspective of neuro-immune regulation, it is our hope to screen out the high-risk population of asthma in early childhood, help find a simpler and more specific monitoring method of asthma treatment, as well as develop new asthma treatments. While for now, more research is expected and this is definitely a field worth further exploration.

## Author Contributions

Study design: CJ and SL. Original draft of manuscript: NZ. Manuscript preparation: NZ and JX. Financial support: CJ and SL. Finalized the manuscript: NZ, CJ, and SL. All authors contributed to the review article and approved the submitted version.

## Funding

We are very grateful for the financial support from the National Natural Science Foundation of China (grant No. 81970029), Shaanxi Province Natural Science Foundation (Project No. 2021JQ-024) and fundamental research funds for the central universities (xjh012020026).

## Conflict of Interest

The authors declare that the research was conducted in the absence of any commercial or financial relationships that could be construed as a potential conflict of interest.

## Publisher’s Note

All claims expressed in this article are solely those of the authors and do not necessarily represent those of their affiliated organizations, or those of the publisher, the editors and the reviewers. Any product that may be evaluated in this article, or claim that may be made by its manufacturer, is not guaranteed or endorsed by the publisher.

## Abbreviations

AHR, airway hyperresponsiveness; Th2, T helper 2; ILC2s, group 2 innate lymphoid cells; TSLP, thymus stromal lymphopoietin; ACh, acetylcholine; ASMs, airway smooth muscle cells; DCs, dendritic cells; CGRP, calcitonin gene-related peptide; PNECs, pulmonary neuroendocrine cells; NT4, neurotrophin 4; OVA, ovalbumin; 5-HT, 5-hydroxytryptamine; TGF-β, transforming growth factor β; β_2_-AR, β2-adrenergic receptor; NGF, nerve growth factor; EETs, eosinophil extracellular traps; NMU, neuromedin U; VIP, vasoactive intestinal peptide.
